# Anti-Glutamic Acid Decarboxylase 65 (Anti-GAD65) Autoimmune Encephalopathy: A Case of Diagnostic and Treatment Challenge

**DOI:** 10.7759/cureus.105312

**Published:** 2026-03-16

**Authors:** Naung Latt Htun, Hsan Theingi Win

**Affiliations:** 1 Internal Medicine, Suri Seri Begawan Hospital, Kuala Belait, BRN

**Keywords:** anti-gad65 encephalitis, anti-gad antibodies, autoimmune encephalopathy, autoimmune limbic encephalitis, multiple autoimmune diseases

## Abstract

Anti-glutamic acid decarboxylase 65 (anti-GAD65) autoimmune encephalopathy is an uncommon but increasingly recognized cause of cognitive impairment and refractory seizures. We report a 69-year-old woman with a background of insulin-treated diabetes mellitus, hypertension, hypercholesterolemia, and prior hyperthyroidism, who presented with acute altered consciousness and cognitive dysfunction. Initial investigations excluded metabolic, infectious, vascular, and paraneoplastic causes. Cerebrospinal fluid (CSF) analysis showed mildly elevated protein without pleocytosis, and MRI brain demonstrated nonspecific periventricular T2/FLAIR hyperintensities. Thyroid antibodies were markedly elevated, raising suspicion for Hashimoto’s encephalopathy; however, an extended autoimmune panel revealed strongly positive anti-GAD65 antibodies (50.9 nmol/L; reference <0.02). She was treated with intravenous immunoglobulin and pulsed methylprednisolone, with partial improvement but persistent slow mentation. Rituximab was planned, but follow-up was lost. This case highlights the diagnostic overlap between anti-GAD65 encephalitis and Hashimoto’s encephalitis, underscores the importance of comprehensive antibody testing in unexplained encephalopathy, and illustrates the incomplete response to first-line immunotherapy.

## Introduction

Autoimmune encephalopathy is an uncommon cause of subacute cognitive decline, seizures, psychiatric symptoms, and altered consciousness. The spectrum of autoimmune encephalitis has expanded significantly with the discovery of neuronal autoantibodies that target intracellular and cell-surface antigens. Among these, antibodies against glutamic acid decarboxylase 65 (GAD65) have emerged as a major cause of autoimmune epilepsy and limbic encephalitis [1]. However, due to non-specific clinical presentations, investigations, and imaging features, the diagnosis remains challenging. Glutamic acid decarboxylase (GAD) is the rate-limiting enzyme that converts glutamate to gamma-aminobutyric acid (GABA), the principal inhibitory neurotransmitter in the central nervous system [2]. While anti-GAD65 antibodies are classically associated with type 1 diabetes mellitus, they are also linked to stiff-person syndrome, cerebellar ataxia, limbic encephalitis, and autoimmune epilepsy [3,4]. High serum titers (≥20 nmol/L) strongly support neurological involvement [1]. Anti-GAD65-associated encephalitis is poorly responsive to first-line immunotherapy, including corticosteroids, intravenous immunoglobulin (IVIG), or plasma exchange. More aggressive or prolonged immunosuppression, such as rituximab or cyclophosphamide, is often required [1,5].

A major diagnostic dilemma arises in patients with concurrent thyroid autoimmunity. Hashimoto’s encephalopathy (HE), also termed steroid-responsive encephalopathy associated with autoimmune thyroiditis (SREAT), is characterized by encephalopathy, seizures, elevated antithyroid antibodies, and a favorable response to corticosteroids [2,6]. Clinical manifestations of HE are highly variable, with normal or mildly elevated CSF protein levels and normal or nonspecific MRI findings [6,7]. Importantly, antithyroid antibodies lack specificity and may be present in other autoimmune neurological conditions [2]. These nonspecific findings overlap considerably with those of anti-GAD65 encephalitis and warrant comprehensive autoimmune antibody testing in cases of unexplained encephalopathy. Given the treatable nature of many autoimmune encephalitides, early recognition is critical. Unlike classic Hashimoto’s encephalopathy, anti-GAD65 encephalitis frequently demonstrates partial or incomplete improvement [1], underscoring the importance of accurate differentiation.

In summary, anti-GAD65 autoimmune encephalopathy represents a rare but important cause of subacute cognitive impairment and seizures, particularly in patients with coexisting autoimmune disease. The overlap with Hashimoto’s encephalopathy and other autoimmune encephalitides poses significant diagnostic challenges. Comprehensive evaluation, including extended neuronal antibody panels, is essential in patients with unexplained encephalopathy, particularly when thyroid antibodies are present, and steroid responsiveness is atypical.

## Case presentation

The patient was a 69-year-old woman with a history of insulin-dependent diabetes, hypertension managed with four antihypertensive medications, and hypercholesterolemia on atorvastatin. She had a brief history of hyperthyroidism in October 2013 and was treated with carbimazole until her thyroid function tests normalized in May 2015. She lived alone and had no complaints before admission. In April 2024, a relative found her lying on the floor and brought her to the hospital. Initial assessment revealed normal blood pressure, heart rate, and oxygen saturation, with a random blood glucose of 17.5 mmol/L and a serum ketone level of 5.5, but normal pH, bicarbonate, and serum osmolality. She had a GCS of 14/15 (E4V5M6) with disorientation to time, place, and person and slow mentation but had no cranial nerve palsies, motor weakness, cerebellar signs, or meningism. She was found to have psoriatic plaque on the trunk and scalp.

The laboratory results showed leukocytosis of 15,500 cells/ml, with a CRP level of 7.59, but normal procalcitonin levels; ESR; electrolytes; renal, liver, and thyroid function tests; as well as Vitamin B12 and folate levels (Table 1).

**Table 1:** Blood and cerebrospinal fluid (CSF) parameters

Parameters	Patient Values	Unit	Reference Range
Hb (Hemoglobin)	12.9	g/dl	11.5 - 15.9
MCV (Mean corpuscular volume)	85	fl	81 - 95.4
WBC (White blood cells)	15.5	x10^3^/uL	4.2 - 12.6
Platelets	341	x10^3^/uL	174 - 430
CRP (C-reactive protein)	7.59	mg/dL	0.00 - 0.5
ESR (Erythrocyte sedimentation rate)	14	mm/hr	3 - 15
Procalcitonin	0.04	ng/mL	< 0.5
Sodium	135	mmol/L	136 - 145
Potassium	4.2	mmol/L	3.5 - 5.1
Bicarbonate	20	mmol/L	22 - 29
Calcium	2.22	mmol/L	2.15 - 2.5
Magnesium	0.74	mmol/L	0.66 - 1.07
Phosphate	0.93	mmol/L	0.81 - 1.45
Uric acid	319	umol/L	142.8 - 339.2
Creatinine	84.9	umol/L	53 - 97.2
Urea	6.2	mmol/L	2.8 - 8.1
Total Bilirubin	20.3	umol/L	0 - 21
ALT (Alanine aminotransferase)	20	U/L	10 - 35
ALP (Alkaline phosphatase)	110	U/L	35 - 104
GGT (Gamma-glutamyl transferase)	35	U/L	5 - 36
Albumin	33	g/L	35 - 52
Total Protein	62	g/L	64 - 83
Glucose Random	14.9	mmol/L	4 - 7.8
Serum Vitamin B12	1181	pmol/L	138 - 652
Serum Folate	10.2	nmol/L	7 - 46.4
Free T3	4.71	pmol/L	3.1 - 6.8
Free T4	16	pmol/L	12 - 22
TSH (Thyroid-stimulating hormone)	1.15	uIU/mL	0.27 - 4.2
8 am Serum Cortisol	358	nmol/L	102.1 - 535.2
HBsAg (Hepatitis B surface antigen)	Nonreactive		
HCV Ab (Hepatitis C virus antibody)	Nonreactive		
HIV Ab (Human immunodeficiency virus antibody)	Nonreactive		
VDRL (Venereal disease research laboratory test)	Nonreactive		
ANA titre (Antinuclear antibody titre)	80		RR <80
ANA Pattern (Profile of antinuclear antibody titre)	Homogeneous		
Anti-DNA Antibodies (ELISA)	<10.00	IU/ml	<100
Extractable Nuclear Antigen			
RNP (Ribonucleoprotein)	Negative		
Sm (Smith antibody)	Negative		
SS-A (Ro)	Negative		
SS-B (La)	Negative		
Scl-70	Negative		
Jo-1	Negative		
PCNA (Proliferating cell nuclear antigen)	Negative		
Ro-52	Negative		
PM-Scl100	Negative		
Centromere B	Negative		
dsDNA	Negative		
Nucleosomes	Negative		
Histones	Negative		
Ribosomal Protein	Negative		
AMA-M2	Negative		
Anti-MPO	<2.0	RU/ml	<20
Anti PR3	<2.0	RU/ml	<20
ANCA-IIFA	Negative		
C3 (Complement C3)	1.33	g/L	0.83 - 1.93
C4 (Complement C4)	0.35	g/L	0.15 - 0.57
Rheumatoid Factor	Negative		Negative: <30 IU/ml
Anti-Thyroid Peroxidase	1253.3	IU/ml	<5.6
Anti-Thyroglobulin	553.9	IU/ml	<4.1
TSH Receptor Antibody	1.4	IU/ml	<3.1
CSF (Cerebrospinal fluid)			
Appearance	Clear and colorless		
WBC (white blood cells)	0		
RBC (red blood cells)	3		
Cryptococcus	No Cryptococcus seen.		
Gram stain	No organism seen.		
Culture and Sensitivity	No growth.		
Glucose	6.6	mmol/L	2 - 5
Protein	0.554	g/L	0.15 - 0.40
AFB Microscopy	No AFB seen.		
AFB Culture	No growth after 8 weeks of incubation.		
Meningitis/ Encephalitis Panel (CSF)			
Escherichia coli K1	Not Detected		Not Detected
Haemophilus influenzae	Not Detected		Not Detected
Listeria monocytogenes	Not Detected		Not Detected
Neisseria meningitidis	Not Detected		Not Detected
Streptococcus agalactiae	Not Detected		Not Detected
Streptococcus pneumoniae	Not Detected		Not Detected
Cytomegalovirus	Not Detected		Not Detected
Enterovirus	Not Detected		Not Detected
Herpes simplex virus 1	Not Detected		Not Detected
Herpes simplex virus 2	Not Detected		Not Detected
Human Herpesvirus 6	Not Detected		Not Detected
Human parechovirus	Not Detected		Not Detected
Varicella zoster virus	Not Detected		Not Detected
Cryptococcus neoformans/gattii	Not Detected		Not Detected
Paraneoplastic Autoimmune Profile (Serum and CSF)			
Anti-Amphiphysin (AmphiAb)	Negative		Negative
Anti-CV2 Antigen (CV2Ab)	Negative		Negative
Anti-Paraneoplastic antigen Ma2 (PNMa2Ab)	Negative		Negative
Anti-Ri (RiAb)	Negative		Negative
Anti-Yo (YoAb)	Negative		Negative
Anti-Hu (HuAb)	Negative		Negative
Anti-Recoverin (RecovAb)	Negative		Negative
Anti-SOX1 (SOX1Ab)	Negative		Negative
Anti-Titin (Titin Ab)	Negative		Negative
Autoimmune Encephalopathy Panel (Serum)			
Alpha-amino-3-hydroxy-5-methyl-4-isoxazolepropionic acid receptor (AMPA-R) Ab	Negative		Negative
Amphiphysin Ab	Negative		Negative
Anti-glial/neuronal nuclear antibody-type 1 (AGNA-1)	Negative		Negative
Antineuronal nuclear antibody-type 1 (ANNA-1)	Negative		Negative
Antineuronal nuclear antibody-type 2 (ANNA-2)	Negative		Negative
Antineuronal nuclear antibody-type 3 (ANNA-3)	Negative		Negative
Contactin-associated protein-like-2 (CASPR2-IgG)	Negative		Negative
Collapsin response-mediator protein-5 neuronal (CRMP-5-IgG)	Negative		Negative
Dipeptidyl-peptidase-like protein-6 (DPPX) Ab	Negative		Negative
Gamma-aminobutyric acid B receptor (GABA-B-R) Ab	Negative		Negative
Glutamic acid decarboxylase (GAD65) Ab Assay	50.9	nmol/L	<0.02
Glial fibrillary acidic protein (GFAP)	Negative		Negative
IgLON family member 5 (IgLON5)	Negative		Negative
Leucine-rich glioma inactivated protein-1 (LGI1-IgG)	Negative		Negative
Metabotropic glutamate receptor 1 (mGluR1) Ab	Negative		Negative
Neurochondrin	Negative		Negative
Neuronal intermediate filament IgG (NIF)	Negative		Negative
N-methyl-D-aspartate-receptor (NMDA-R) Ab	Negative		Negative
Purkinje cell cytoplasmic antibody, type 1 (PCA-1)	Negative		Negative
Purkinje cell cytoplasmic antibody, type 2 (PCA-2)	Negative		Negative
Purkinje cell cytoplasmic antibody, type Tr (PCA-Tr)	Negative		Negative
Septin-7	Negative		Negative

**Figure 1:**
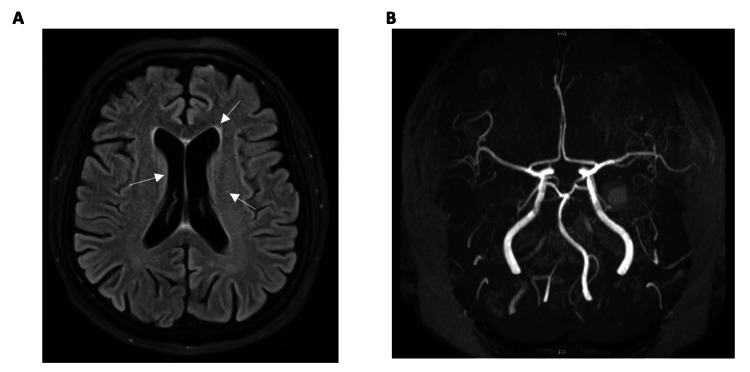
Magnetic resonance imaging (MRI) of the brain showed a few discrete hyperintensities in the bilateral periventricular region (A), with unremarkable findings on the magnetic resonance angiogram (MRA) (B)

Blood and urine cultures were negative for growth. A normal CT scan of the brain ruled out an acute stroke. The CSF examination revealed clear fluid with a glucose of 6.6 mmol/L (normal range 2-5 mmol/L), slightly elevated protein of 0.554 g/L (normal 0.15-0.4 g/L), WCC of 0 cells/uL, RBC of 3 cells/uL, with a negative culture and a negative meningitis/encephalitis panel for various viruses and bacteria, AFB, and paraneoplastic autoimmune profile. Serology for Hepatitis B, C, HIV, and syphilis was non-reactive. The ANA titer was 1:80 with a homogeneous pattern, but anti-dsDNA antibodies, ENA, rheumatoid factor, complement, and ANCA were negative. Thyroid antibodies were positive for anti-thyroid peroxidase at 1253.3 IU/ml (<5.6) and anti-thyroglobulin at 553.9 IU/ml (<4.1), with a normal TSH receptor antibody. MRI of the brain revealed a few discrete T2/FLAIR hyperintensities in the bilateral periventricular regions and centrum semiovale, without diffusion abnormalities, and an unremarkable MRA (Figure 1).

The EEG showed diffuse mild-to-moderate slowing of background activity, indicative of a mild-to-moderate non-specific encephalopathy, and a single episode of sharp activity in the right temporal region, which was of undetermined significance. The extended autoimmune encephalopathy panel was negative except for GAD65 Ab, which was 50.9 nmol/L (<0.02). Initially, she was considered to have Hashimoto’s encephalopathy, but the strongly positive anti-GAD65 Ab prompted a diagnosis of anti-GAD65 autoimmune encephalitis. She was treated with IV immunoglobulin at 0.4 g/kg daily for five days and IV pulse methylprednisolone at 500 mg every two weeks for two months. Although high-dose intravenous methylprednisolone at 1000 mg daily for 3-5 days is frequently reported in autoimmune encephalitis, a lower pulsed dose of 500 mg was chosen in this patient due to her underlying co-morbidities, such as brittle diabetes and resistant hypertension, which increase the risk of steroid-related complications. After treatment, her mini-mental state examination (MMSE) [8] was 17/30 compared to 16/30 before starting treatment, showing persistent cognitive impairment. She was scheduled to receive rituximab, but did not attend the follow-up appointment.

## Discussion

This case highlights several key diagnostic and therapeutic challenges in anti-GAD65 autoimmune encephalopathy, especially when coexisting with thyroid autoimmunity. The patient exhibited acute altered consciousness and ongoing cognitive impairment, with markedly elevated anti-thyroid antibodies initially indicating Hashimoto’s encephalopathy. However, strongly positive anti-GAD65 antibodies ultimately confirmed the diagnosis of GAD65-associated autoimmune encephalitis. Clinical features of HE are diverse and may include cognitive impairment, psychiatric symptoms, ataxia, myoclonus, and seizures [7]. Nevertheless, antithyroid antibodies are not disease-specific and are also present in other autoimmune encephalitides [2]. Case reports have shown that anti-TPO antibodies can be present in anti-GAD65 encephalitis and other autoimmune conditions, and that HE remains a diagnosis of exclusion [2]. In our case, although thyroid antibodies were significantly elevated, the absence of a dramatic steroid response and the presence of high-titer anti-GAD65 antibodies were consistent with a GAD65-mediated disease. Several reports have shown that HE may present with normal or nonspecific MRI findings [7], as in our patient, who had discrete periventricular T2/FLAIR hyperintensities. Furthermore, CSF findings in HE may show mild protein elevation or be normal [7], which again overlaps with our findings. This significant clinical and investigative overlap underscores the need for extended neuronal antibody panels in cases of unexplained encephalopathy.

Anti-GAD65 antibodies are strongly associated with neurological syndromes when present at high titers. In autoimmune epilepsy and encephalitis, serum titers ≥20 nmol/L are considered supportive of neurological involvement [1]. Our patient’s level of 50.9 nmol/L is well above this threshold, strengthening the diagnostic conclusion. Anti-GAD65 antibodies are most commonly linked to temporal lobe epilepsy and limbic encephalitis. However, the clinical spectrum is broader and may include subacute cognitive decline and encephalopathy without prominent seizures [3]. Our patient’s persistent cognitive impairment despite IVIG and pulse corticosteroids aligns with the observation that anti-GAD65 syndromes are often only moderately responsive to first-line immunotherapy.

Unlike cell-surface antibody-mediated encephalitis (e.g., NMDA receptor), anti-GAD65 targets an intracellular antigen. Intracellular antigen-mediated syndromes are thought to involve cytotoxic T-cell-mediated neuronal injury rather than direct antibody pathogenicity [1]. In autoimmune epilepsy, intracellular antigen-associated disorders generally have a poorer response to immunotherapy compared to cell-surface antigen-associated syndromes [9]. This mechanism likely explains the incomplete cognitive recovery observed in our patient. It also highlights the critical importance of early recognition before irreversible neuronal injury develops. Anti-GAD65-associated epilepsy and encephalitis frequently show limited response to corticosteroids, IVIG, or plasma exchange [1]. More aggressive immunosuppressive therapies, including rituximab or cyclophosphamide, are often required [1,3,5]. 

In contrast, HE is typically characterized by good steroid responsiveness [6]. In our patient, IVIG and pulse methylprednisolone stabilized the clinical course but did not significantly improve cognitive deficits. This partial response is consistent with the literature on anti-GAD65 encephalitis. Rituximab was appropriately planned as second-line therapy; however, loss to follow-up prevented assessment of long-term outcome.

This case emphasizes several learning points: elevated thyroid antibodies should not automatically lead to a diagnosis of Hashimoto’s encephalopathy, high-titer anti-GAD65 antibodies strongly support neurological autoimmunity, MRI and CSF findings are frequently nonspecific in both HE and anti-GAD65 encephalitis, and intracellular antigen-associated syndromes are typically more refractory to treatment than cell-surface antibody-associated disorders. Given the increasing recognition of autoimmune encephalitis, clinicians should maintain a high index of suspicion in patients presenting with subacute encephalopathy, especially when coexisting autoimmune conditions such as diabetes mellitus, psoriasis, or thyroid autoimmunity are present.

## Conclusions

In summary, this case highlights the diagnostic complexity of anti-GAD65 autoimmune encephalopathy in the setting of concurrent thyroid autoimmunity. The markedly elevated anti-GAD65 titer, partial steroid responsiveness, and persistent cognitive deficits favor GAD65-mediated encephalitis over classic Hashimoto’s encephalopathy. Comprehensive neuronal antibody testing is essential in unexplained encephalopathy, and early escalation of immunotherapy may be required to prevent long-term neurological sequelae.
